# Studies of Halogen Bonding Induced by Pentafluorosulfanyl Aryl Iodides: A Potential Group of Halogen Bond Donors in a Rational Drug Design

**DOI:** 10.3390/molecules24193610

**Published:** 2019-10-07

**Authors:** Yuji Sumii, Kenta Sasaki, Seiji Tsuzuki, Norio Shibata

**Affiliations:** 1 Department of Nanopharmaceutical Sciences, and Department of Life Science and Applied Chemistry, Nagoya Institute of Technology, Gokiso, Showa-ku, Nagoya 466-8555, Japan; sumii.yuji@nitech.ac.jp (Y.S.); k.sasaki.699@stn.nitech.ac.jp (K.S.); 2 Research Center for Computational Design of Advanced Functional Materials, AIST, Tsukuba, Ibaraki 305-8568, Japan; s.tsuzuki@aist.go.jp; 3 Institute of Advanced Fluorine-Containing Materials, Zhejiang Normal University, 688 Yingbin Avenue, Jinhua 321004, China

**Keywords:** halogen bonding, fluorine, iodine, pentafluorosulfanyl, titration, ab initio calculation, NMR study, drug design

## Abstract

The activation of halogen bonding by the substitution of the pentafluoro-λ^6^-sulfanyl (SF_5_) group was studied using a series of SF_5_-substituted iodobenzenes. The simulated electrostatic potential values of SF_5_-substituted iodobenzenes, the ab initio molecular orbital calculations of intermolecular interactions of SF_5_-substituted iodobenzenes with pyridine, and the ^13^C-NMR titration experiments of SF_5_-substituted iodobenzenes in the presence of pyridine or tetra (*n*-butyl) ammonium chloride (TBAC) indicated the obvious activation of halogen bonding, although this was highly dependent on the position of SF_5_-substitution on the benzene ring. It was found that 3,5-bis-SF_5_-iodobenzene was the most effective halogen bond donor, followed by *o*-SF_5_-substituted iodobenzene, while the *m*- and *p*-SF_5_ substitutions did not activate the halogen bonding of iodobenzenes. The similar *ortho*-effect was also confirmed by studies using a series of nitro (NO_2_)-substituted iodobenzenes. These observations are in good agreement with the corresponding Mulliken charge of iodine. The 2:1 halogen bonding complex of 3,5-bis-SF_5_-iodobenzene and 1,4-diazabicyclo[2.2.2]octane (DABCO) was also confirmed. Since SF_5_-containing compounds have emerged as promising novel pharmaceutical and agrochemical candidates, the 3,5-bis-SF_5_-iodobenzene unit may be an attractive fragment of rational drug design capable of halogen bonding with biomolecules.

## 1. Introduction

**Figure 1:**
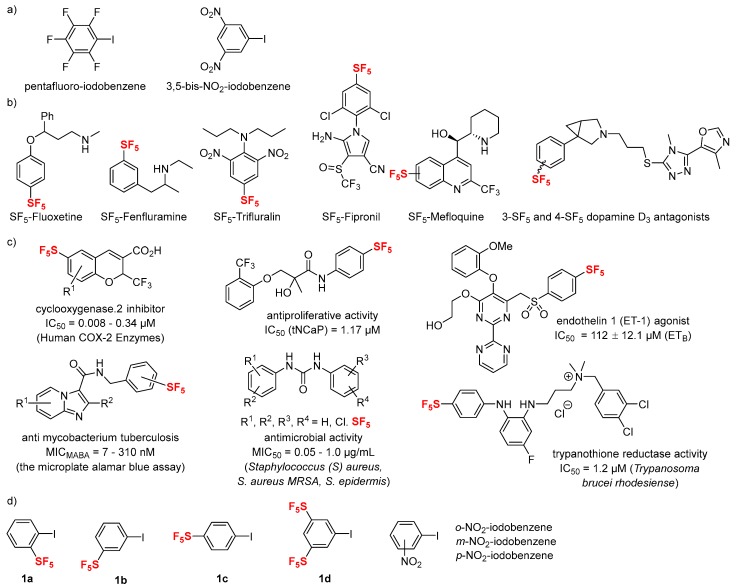
(**a**) The standard halogen bond donors pentafluoro-iodobenzene and 3,5-bis-NO_2_-iodobenzene. (**b**) Examples of pentafluoro-λ^6^-sulfanyl (SF_5_)-containing analogs of marketed drugs. (**c**) Examples of SF_5_-containing biologically active molecules. (**d**) Potential halogen bond donors of aryl iodide containing SF_5_-group(s) and NO_2_-iodobenzenes (this work).

Halogen bonding has attracted considerable attention in recent decades [1,2,3,4], particularly after the pioneering work on halogen bonding in supramolecular chemistry by Resnati et al. [5]. The application of halogen bonding has expanded to a wide variety of fields including crystal engineering, supramolecular assemblies, liquid crystals, rational drug design, and organic reactions [1,2,3,4,5]. Halogen bonding is a noncovalent attraction between an electron-deficient region of a halogen atom (σ-hole, a halogen bond donor) and an electron-rich center of molecules such as nitrogen, oxygen, and sulfur (Lewis base, a halogen bond acceptor). The strength of the halogen bond increases with an increase of the positive electrostatic potential of the σ-hole, which can be activated by substitution of an electron-withdrawing group in the neighborhood of the halogen atom. Thus, perfluoroalkyl iodides and perfluoro-iodobenzenes are well-studied halogen bond donors. In particular, aromatic iodides are of great interest due to the design of halogen bond donors activated by substitution with several electron-withdrawing substituents [6,7]; pentafluoro-iodobenzene [8,9] and 3,5-bis-nitro(NO_2_)-iodobenzene [10] are representative examples (Figure 1a). Besides, in these planar halogen bond donors, the intermolecular π−π charge-transfer [11], anion–π [12,13], cation–π [14,15], and lone pair–π [16,17,18,19] interactions are always competition for and/or form a combination with halogen bonding in molecular assemblies. These aspects make the design of halogen bond donors more elaborate, especially for applications in the rational design of drugs [20]. Statistical analysis of the protein structure database (PDB) showed that a noncovalent interaction between halogenated ligands (halogen-containing drugs, halogen bond donors) and proteins (halogen bond acceptors) frequently contributes to increasing selectivity and binding affinity [21,22]. This survey revealed that a potential rational drug design is possible when the focus is on halogen bonding interactions of halogenated drug candidates and nitrogen, oxygen, sulfur, and phosphate groups on biomolecules such as peptides, protein, and DNA [1,23]. 

In this context, we became interested in pentafluoro-λ^6^-sulfanyl (SF_5_)-substituted iodobenzenes (**1**) as a new group of halogen bond donors. The SF_5_ group has attracted much attention in the field of pharmaceuticals and agrochemicals [24,25,26,27,28,29]. Given the impressive physiochemical properties of the SF_5_ unit, which include its high electronegativity (σ*_m_* = 0.61, σ*_p_* = 0.68; nearly equivalent to the NO_2_ group: σ*_m_* = 0.73, σ*_p_* = 0.78) [30,31,32], high lipophilicity (π = 1.51; greater than that of the CF_3_ group: π = 0.88 and the NO_2_ group: π = −0.28) [32,33,34], and steric hindrance (nearly equivalent to that of the *tert*-butyl group) [34,35], the SF_5_-containing analogs of marketed drugs are attractive candidates in the future drug market (Figure 1b) [36,37,38,39,40,41]. More and more examples of biologically active SF_5_-containing drug candidates have been reported in recent years (Figure 1c) [35,38,39,40,41,42,43,44]. Extending our research to the design and synthesis of SF_5_-containing biologically attractive molecules [45,46,47,48,49,50,51,52,53,54,55,56,57] and a halogen bonding research program [58,59], we are interested in aryl iodides **1a**–**d** consisting of SF_5_-group(s) in the benzene ring as potential drug fragments capable of halogen bonding, in particular, 3,5-bis-SF_5_-iodobenzene (**1d**, Figure 1d). In this research, we have examined the halogen bonding induced by SF_5_-aryl iodides **1a**–**d**. The NO_2_-substituted iodobenzenes pentafluoro-iodobenzene and iodobenzene were also examined for comparisons.

## 2. Results and Discussion

**Figure 2:**
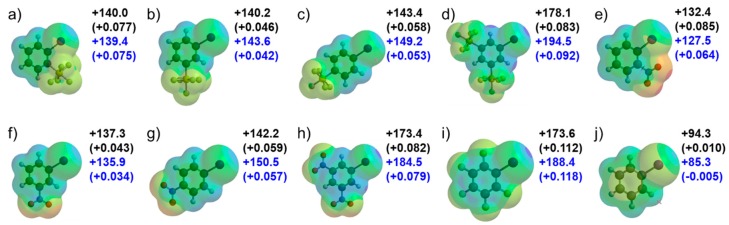
The electrostatic potential surface of molecules with maximum values at iodine atoms in a vacuum (black) and in water (blue). The numbers indicated interaction energy (in kJ/mol) and Mulliken charge in parenthesis; (**a**) *o*-SF_5_-iodobenzene (**1a**), (**b**) *m*-SF_5_-iodobenzene (**1b**), (**c**) *p*-SF_5_-iodobenzene (**1c**), (**d**) 3,5-bis-SF_5_-iodobenzene (**1d**), (**e**) *o*-NO_2_-iodobenzene, (**f**) *m*-NO_2_-iodobenzene, (**g**) *p*-NO_2_-iodobenzene, (**h**) 3,5-bis-NO_2_-iodobenzene, (**i**) pentafluoro-iodobenzene, and (**j**) iodobenzene.

The preparation of *o*-, *m*-, *p*-SF_5_-iodobenzenes (**1a**–**c**), and 3,5-bis-SF_5_-iodobenzene (**1d**) was achieved by the copper-catalyzed halogen exchange reaction of corresponding aryl-bromides according to our previous report [48,60]. We first simulated the electrostatic potential values for the iodine atoms of the targeted SF_5_-iodobenzenes, along with the corresponding values for NO_2_-iodobenzenes, pentafluoro-iodobenzene, and iodobenzene for comparisons. Molecular electrostatic potential surfaces of iodobenzenes were calculated with the density functional Becke, 3-parameter, Lee–Yang–Parr (B3LYP) level of theory with a 6-311++G** basis set in a vacuum and water according to the reported method [61] (Figure 2). The numbers indicate the molecular electrostatic potential (MEP, kJ/mol) between the positive point probe and the surface of the molecule at that particular point, and Mulliken charges at the iodine atom. The MEP value indicates a positive surface potential. The results disclose that **1d** shows a more positive electrostatic potential value and Mulliken charge (Figure 2d), almost as same as that of the well-known halogen bond donors 3,5-bis-NO_2_-iodobenzene (Figure 2h) [10] and pentafluoro-iodobenzene (Figure 2i), independent of calculation in a vacuum and water. It should be noted that *m*- and *p*-substituted SF_5_-iodobenzenes (**1b**,**1c**, respectively) had a lower Mulliken charge of the iodine atom compared to *o*-SF_5_-iodobenzene (**1a**), indicating a poor ability for halogen bonding (Figure 2a vs. Figure 2b,c). The similar tendency of lower Mulliken charge was also observed for *m*- and *p*-substituted NO_2_-iodobenzenes, while *o*-substituted NO_2_-iodobenzene has a more positive Mulliken charge (Figure 2e vs. Figure 2f,g). Iodobenzene had the most negative value and was thus most de-activated (Figure 2j). 

**Figure 3:**
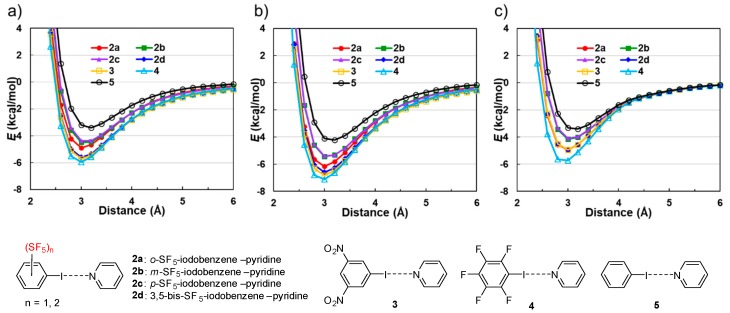
Interaction energy curves (energies versus I---N distance (*R*)) calculated for the iodobenzene---pyridine complexes (**2**–**5**). (**a**) second order Møller–Plesset perturbation calculations (MP2; vacuum), (**b**) Becke, 3-parameter, Lee–Yang–Parr calculation with Grimme’s D3 dispersion correction (B3LYP-D3, vacuum), and (**c**) B3LYP-D3 (water).

Next, the intermolecular interactions of iodobenzenes and pyridine were investigated by ab initio molecular orbital calculations. The intermolecular interaction energies [12,62,63] for the *o*-, *m*-, and *p*-SF_5_-iodobenzene---pyridine complexes (**2a**–**c**), the 3,5-bis-SF_5_-iodobenzene---pyridine complex (**2d**), the 3,5-bis-NO_2_-iodobenzene---pyridine complex (**3**), and the pentafluoro-iodobenzene---pyridine complex (**4**) were calculated by changing the intermolecular separation in a vacuum using the second order Møller–Plesset perturbation method and the cc-pVTZ basis set (MP2/cc-pVTZ). The interaction energy potentials calculated for the complexes were compared with the interaction energy potential calculated for the iodobenzene---pyridine complex (**5**) (Figure 3a). The depths of interaction energy potentials of the SF_5_-iodobenzene---pyridine complex (**2a–d**) are deeper than those of the iodobenzene---pyridine complex (**5**). We note that the potential for *o*-SF_5_-substituted **2a** is much deeper than for *m*-SF_5_-**2b** and *p*-SF_5_-**2c**, while the potential for 3,5-bis-SF_5_-**2d** is much deeper. The depths of interaction energy potentials of 3,5-bis-SF_5_-**2d** and 3,5-bis-NO_2_-**3** are almost the same, and that of the pentafluoro-iodobenzene---pyridine complex (**4**) is the deepest. The interaction energies by the coupled-cluster calculations with single and double substitutions with non-iterative triple excitations [CCSD(T)] at the basis set limit (*E*_CCSD(T)(limit)_) for the optimized geometries of **2a–d**, **3**, **4**, and **5** were calculated. [64] The *E*_CCSD(T)(limit)_ and the contribution of each intermolecular force are summarized in Table 1. The calculations show that the electrostatic (*E*_es_) and dispersion (*E*_corr_) interactions are the primary sources of the attraction, and the substituent dependence of the electrostatic interactions is mainly responsible for the substituent effects on the magnitude of the attraction of the halogen bonds. These calculated results strongly indicate that substitution of the SF_5_ group induces halogen bonding, whose strength depends on the position of the substitution and numbers. The interaction energy potentials were also calculated by the density functional theory (DFT) method (B3LYP functionals with Grimme’s D3 dispersion correction [65]) with and without polarizable continuum model (PCM) [66] to evaluate the effects of water as shown in Figure 3b,c. The depth of the potential calculated for each complex in water is 20–30% smaller than that calculated in a vacuum. As expected, 3,5-bis-SF_5_-iodobenzene (**1d**) is the most efficient template to induce halogen bonding in SF_5_-iodobenzenes (**1**), equivalent to the well-known halogen bond donor 3,5-bis-NO_2_-iodobenzene. The 3,5-bis-substitution is also attractive for improved biological activity, as evidenced in medicinal research [45,60]. 

**Figure 4:**
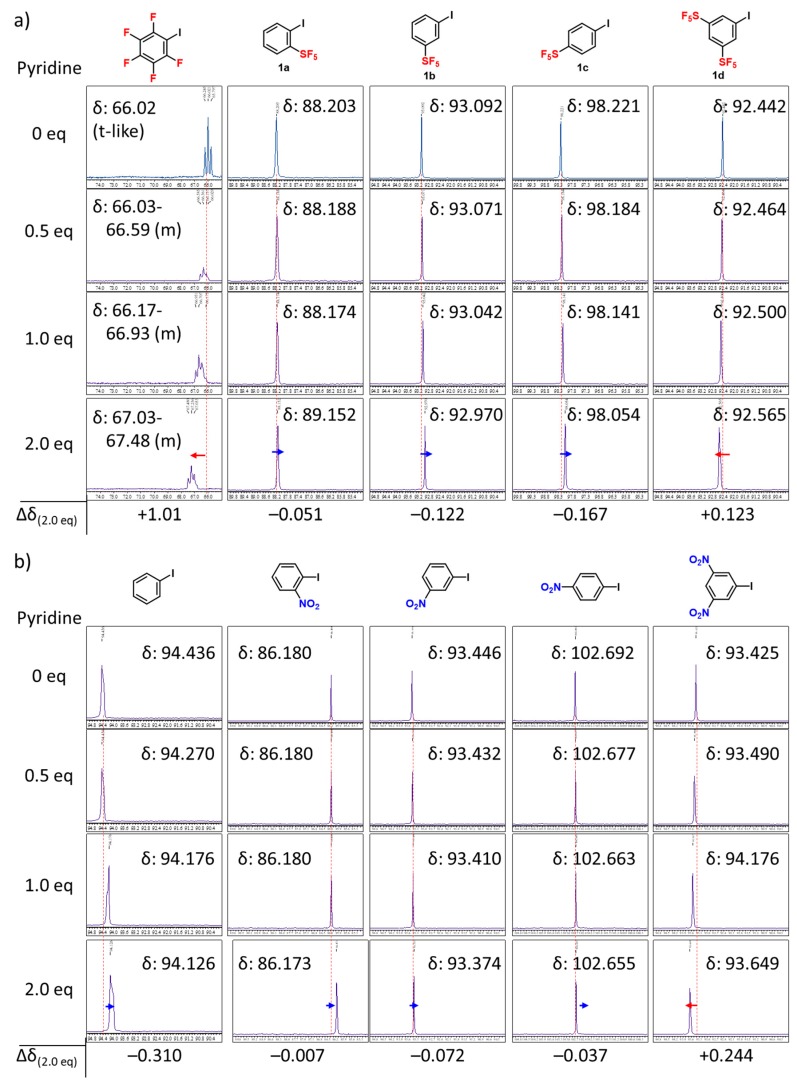
Change in the ^13^C-NMR chemical shift of the carbon atom bonded to iodine in Ar−I with equivalents of pyridine in CDCl_3_. (**a**) pentafluoro-iodobenzene; *o*-SF_5_-iodobenzene (**1a**); *m*-SF_5_-iodobenzene (**1b**); *p*-SF_5_-iodobenzene (**1c**); 3,5-bis-SF_5_-iodobenzene (**1d**). (**b**) iodobenzene; *o*-NO_2_-iodobenzene; *m*-NO_2_-iodobenzene; *p*-NO_2_-iodobenzene; 3,5-bis-NO_2_-iodobenzene.

**Figure 5:**
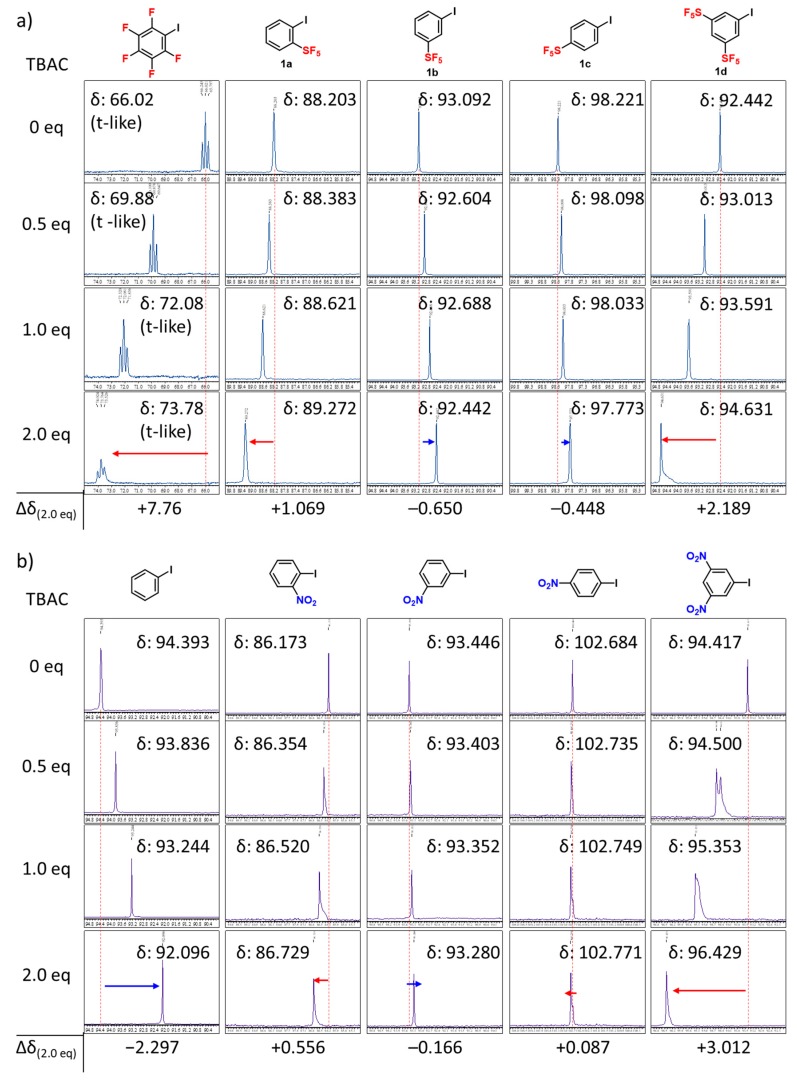
Change in the ^13^C-NMR chemical shift of the carbon atom bonded to iodine in Ar−I with equivalents of tetra (*n*-butyl) ammonium chloride (TBAC) in CDCl_3_. (**a**) pentafluoro-iodobenzene; *o*-SF_5_-iodobenzene (**1a**); *m*-SF_5_-iodobenzene (**1b**); *p*-SF_5_-iodobenzene (**1c**); 3,5-bis-SF_5_-iodobenzene (**1d**). (**b**) iodobenzene; *o*-NO_2_-iodobenzene; *m*-NO_2_-iodobenzene; *p*-NO_2_-iodobenzene; 3,5-bis-NO_2_-iodobenzene.

Encouraged by the results of these calculations, ^13^C-NMR titration experiments of SF_5_-iodobenzenes in the presence of pyridine or tetra(*n*-butyl)ammonium chloride (TBAC) in CDCl_3_ to detect the existence of halogen bonding were carried out with comparisons using pentafluoro-iodobenzene and iodobenzene. ^13^C-NMR is a well-studied technique to probe halogen bonding [67,68,69,70]. Namely, the increase of chemical shifts of the carbon atom bonded to iodine in Ar−I indicates stronger halogen bonding due to lengthening of the carbon−iodine bond by the donation of electrons from the halogen bond acceptor into iodine orbitals [12,71]. Our ^13^C-NMR experiments are shown in Figure 4 and Figure 5. First, the chemical shift of the carbon atom bonded to iodine in pentafluoro-iodobenzene increased with an increase in the addition of pyridine or TBAC. This phenomenon confirms the formation of halogen bonding interaction on the σ-hole of the iodine atom with a Lewis base (nitrogen atom of pyridine, or Cl anion). The observed up-field shift is a consequence of the donation of electron density from the halogen bond acceptor to the iodine group, and the more significant shifts donated by TBAC with respect to pyridine are consistent with the fact that anions function as better electron donors than pyridines, and form a stronger halogen bond than the nitrogen atom which possesses a neutral lone-pair.

On the other hand, the addition of pyridine or TBAC to iodobenzene leads to a decrease of the chemical shift of the carbon atom bonded to the iodine atom. This observation could be explained by the competitive interaction of the intermolecular π–π interaction [72] (with pyridine) or the cation–π interaction [73] (with tetra (*n*-butyl) ammonium cation), although this explanation needs further experimental support. Nevertheless, pentafluoro-iodobenzene is an activated halogen bond donor, but iodobenzene is not.

We next examined the titration experiments of a series of SF_5_-substituted iodobenzenes (**1a**–**d**) (Figure 4a and Figure 5a). As mentioned above, the changes in chemical shift of pyridine titration are much smaller than those of TBAC titration, while the occurrence of halogen bonding is fundamentally the same. The chemical shifts of the carbon atom bonded to iodine in *o*-SF_5_-iodobenzene (**1a**) and 3,5-bis-SF_5_-iodobenzene (**1d**) increased after the addition of TBAC, which confirms the occurrence of halogen bonding. On the other hand, opposite phenomena were observed in the case of *m*- and *p*-SF_5_-iodobenzene (**1b**, **1c**, respectively) with TBAC, which confirms the absence of halogen bonding. These results of the occurrence/absence of halogen bonding depend on the *o*-, *m*-, or *p*-position of SF_5_-substitution, and are in good agreement with the Mulliken charges, as shown in Figure 2. This *ortho*-effect was also observed for the titration experiments of a series of NO_2_-substituted iodobenzenes (Figure 4b and Figure 5b), which are in good agreement with the calculated Mulliken charges, as shown in Figure 2.

**Figure 6:**
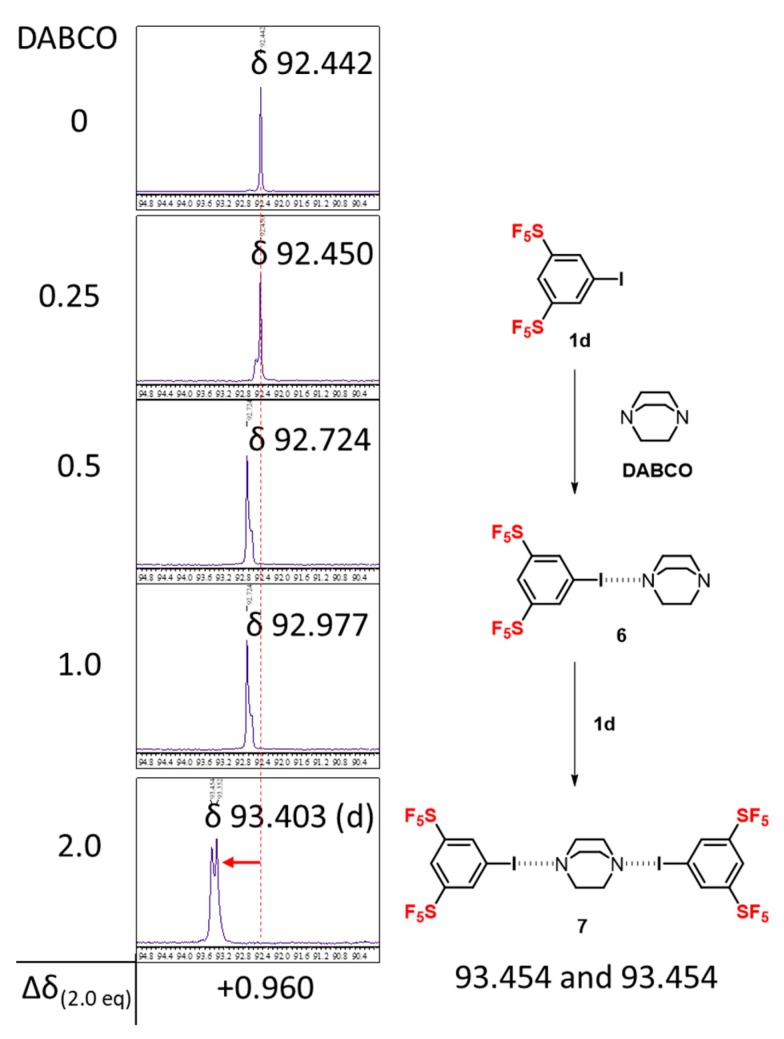
Change in the ^13^C-NMR chemical shift of the carbon atom bonded to iodine in **1d** with equivalents of 1,4-diazabicyclo[2.2.2]octane (DABCO) in CDCl_3_.

Finally, we examined the formation of halogen bonding interaction between 3,5-bis-SF_5_-iodobenzene (**1d**) and 1,4-diazabicyclo[2.2.2]octane (DABCO). Since DABCO is a bifunctional halogen bond donor, the 2:1 halogen bonding complex of **1d** and DABCO is expected. Indeed, X-ray crystallographic analysis of 3,5-bis-NO_2_-iodobenzene with DABCO revealed the formation of a 2:1 halogen bonding complex [10]. We thus examined the titration of **1d** by the addition of DABCO (Figure 6). With an increasing amount of DABCO, the chemical shift of carbon attached to iodine increased, providing proof of the formation of halogen bonding of I---N. More interestingly, one singlet peak at 92.442 ppm gradually changed to double singlets at 93.454 and 93.352 ppm, which provides evidence of two halogen bonds in the complex from **6** to **7**. This phenomenon suggests that the 2:1-complex **7** is not entirely symmetrical, as in the case of 3,5-bis-NO_2_-iodobenzene [10]. 

## 3. Materials and Methods

All reactions were performed in oven-dried glassware under a positive pressure of nitrogen. Solvents were transferred via syringe and were introduced into the reaction vessels through a rubber septum. Chemicals were purchased and used without further purification unless otherwise noted. All of the reactions were monitored by thin-layer chromatography (TLC) carried out on a 0.25 mm Merck silica gel (60-F_254_) (Kenilworth, NJ, USA). TLC plates were visualized with UV light and 7% phosphomolybdic acid or KMnO_4_ in water/heat. Column chromatography was carried out on a column packed with silica gel (60N spherical neutral size 50–60 μm) supplied by Kanto Chemical Co., Inc. (Tokyo, Japan). The ^1^H-NMR (300 MHz), ^19^F-NMR (282 MHz), and ^13^C-NMR (126 MHz) spectra for each solution in CDCl_3_ were recorded on Varian Mercury 300 (Agilent Technologies, Palo Alto, CA, USA) and Avance 500 (Bruker, Billerica, MA, USA) NMR spectrometers. Chemical shifts (δ) are expressed in ppm downfield from internal tetramethylsilane (δ = 0.0 ppm) for ^1^H-NMR, C_6_F_6_ (δ = −162.2 ppm) for ^19^F-NMR, and CDCl_3_ (δ = 77.00 ppm) for ^13^C-NMR. Mass spectrometry was recorded on a SHIMADZU LCMS-2020 (ESI-MS) (Shimadzu Corporation, Kyoto, Japan). The ^1^H, ^13^C and ^19^F-NMR spectra of compounds 1 are available in the Supplementary Material.

### 3.1. General Procedure of Pentafluoro-λ^6^-Sulfanyl Iodobenzene


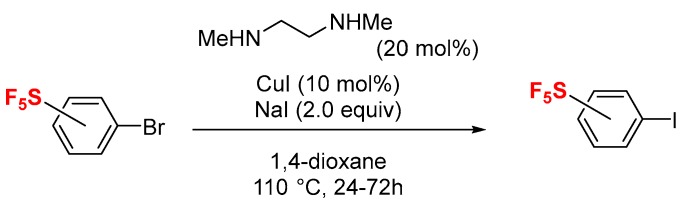


*o*-, *m*-, *p*-SF_5_-iodobenzenes (**1a**–**c**), and 3,5-bis-SF_5_-iodobenzene (**1d**) were prepared from corresponding aryl bromides by a copper-catalyzed halogen exchange reaction. [74]. To a flame-dried Schlenk-tube, CuI (10 mol %), NaI (2.0 equiv), and aryl bromide (1.0 equiv) were added and evacuated and backfilled with argon. 1,4-Dioxane (1.0 mL/mmol for ArBr) and *N*,*N*′-dimethylethylenediamine (20 mol %) were added; the mixture was stirred at room temperature for 3 min and then at 110 °C for 24–72 h. The resulting suspension was cooled to room temperature and filtered through a pad of SiO_2_. The filtrate was diluted in NaHCO_3_ aq and extracted with CH_2_Cl_2_ three times. The combined organic layer was washed with brine, dried over Na_2_SO_4_, and concentrated under reduced pressure. The crude product was purified by column chromatography on silica gel (*n*-hexane) to give the desired product.

#### 3.1.1. Pentafluoro(2-iodophenyl)-λ^6^-sulfane (**1a**)

92% yield. ^1^H-NMR (300 MHz, CDCl_3_) δ 8.14 (d, *J* = 7.6 Hz, 1H), 7.81 (d, *J* = 8.5 Hz, 1H), 7.45 (t-like, *J* = 7.5 Hz, 1H), 7.12 (t-like, *J* = 7.4 Hz, 1H). ^19^F-NMR (282 MHz, CDCl_3_) δ: 83.5 (quintet, *J* = 152.6 Hz, 1F), 63.5 (d, *J* = 151.7 Hz, 4F) ppm. ^13^C-NMR (126 MHz, CDCl_3_) δ 158.4 (quintet, *J* = 16.3 Hz), 143.9, 132.2, 130.1 (t-like, *J* = 5.4 Hz), 127.9, 88.2 ppm. MS (ESI) *m*/*z*: 353 [(M + Na)^+^]. The product was consistent with previously reported characterization data [60].

#### 3.1.2. Pentafluoro(3-iodophenyl)-λ^6^-sulfane (**1b**) 

91% yield. ^1^H-NMR (300 MHz, CDCl_3_) δ 8.08 (s, 1H), 7.86 (d, *J* = 7.6 Hz, 1H), 7.74 (d, *J* = 7.4 Hz, 1H), 7.22 (t-like, *J* = 8.4 Hz, 1H) ppm. ^19^F-NMR (282 MHz, CDCl_3_) δ 82.5 (quintet, *J* = 150.9 Hz, 1F), 62.3 (d, *J* = 151.7 Hz, 4F) ppm. ^13^C-NMR (126 MHz, CDCl_3_) δ 154.4 (quintet, *J* = 17.7 Hz), 140.6, 134.7 (t-like, *J* = 4.5 Hz), 130.3, 125.2, 93.1 ppm. MS (ESI) *m*/*z*: 353 [(M + Na)^+^]. The product was consistent with previously reported characterization data [48,75,76,77].

#### 3.1.3. Pentafluoro(4-iodophenyl)-λ^6^-sulfane (**1c**) 

91% yield. ^1^H-NMR (300 MHz, CDCl_3_) δ 7.82 (d, *J* = 8.2 Hz, 2H), 7.48 (d, *J* = 8.5 Hz, 2H) ppm. ^19^F-NMR (282 MHz, CDCl_3_) δ 83.0 (quintet, *J* = 150.0 Hz, 1F), 62.3 (d, *J* = 150.0 Hz, 4F) ppm. ^13^C-NMR (126 MHz, CDCl_3_) δ 153.5 (quintet, *J* = 18.2 Hz), 137.9, 127.5 (t-like, *J* = 4.5 Hz), 98.2 ppm. MS (ESI) *m*/*z*: 353 [(M + Na)^+^]. The product was consistent with previously reported characterization data [48,76,77].

#### 3.1.4. (5-Iodo-1,3-phenylene)bis(pentafluoro-λ^6^-sulfane) (**1d**)

95% yield. ^1^H-NMR (300 MHz, CDCl_3_) δ 8.23 (s, 1H), 8.12–8.07 (m, 2H) ppm. ^19^F-NMR (282 MHz, CDCl_3_) δ 80.1 (quintet, *J* = 151.7 Hz, 1F), 62.7 (d, *J* = 151.7 Hz, 4F) ppm. ^13^C-NMR (126 MHz, CDCl_3_) δ 154.0 (quintet, *J* = 20.0 Hz), 137.8, 123.5 (t, *J* = 4.5 Hz), 92.4 ppm. MS (ESI) *m*/*z*: 479 [(M + Na)^+^]. The product was consistent with previously reported characterization data [48].

### 3.2. Computational Method

#### 3.2.1. Calculations for Electrostatic Potential Values

Molecular electrostatic potential surfaces for SF_5_ or NO_2_-iodobenzenes were calculated with the density functional B3LYP level of theory with 6-311++G** basis set in a vacuum or in a water. The same level of theory was used to calculate the electrostatic potentials with the results presented in Figure 2. All calculations were carried out using Spartan′18 software (Wavefunction, Inc., Irvine, CA, USA). All molecules were geometry optimized with the maxima and minima in the electrostatic potential surface (0.002 e/au isosurface) determined using a positive point charge in the vacuum as a probe. The numbers indicate the interaction energy (kJ/mol) between the positive point probe and the surface of the molecule at that particular point. A positive value for the interaction energy indicates a positive surface potential.

#### 3.2.2. Calculations for Interaction Energies

The Gaussian 09 program [78] was used for the ab initio molecular orbital calculations. The basis sets implemented in the program were used. Electron correlation was accounted for by the second-order Møller–Plesset perturbation (MP2) method [79,80], Becke, 3-parameter, Lee–Yang–Parr (B3LYP) [81,82,83], and by coupled-cluster calculations with single and double substitutions with non-iterative triple excitations (CCSD(T)) [64]. The basis-set superposition error (BSSE) [84] was corrected for all calculations by using the counterpoise method unless otherwise noted [85]. The geometries of the complexes were optimized at the counterpoise-corrected MP2/6-311G* level. The DGDZVP basis set [86] was used for iodide. The MP2 interaction energies of the complexes at the basis set limit (*E*_MP2(limit)_) were estimated by the method of Helgaker et al. [87] from the calculated MP2 interaction energies (*E*_MP2_) by using the aug-cc-pVDZ and aug-cc-pVTZ basis sets. The *E*_MP2(limit)_ for the 3,5-bis-NO_2_-iodobenzene---pyridine complex (**3**) was estimated using the cc-pVDZ and cc-pVTZ basis set. The CCSD(T) interaction energies at the basis set limit (*E*_CCSD(T)(limit)_) were calculated as the sum of *E*_MP2(limit)_ and the estimated CCSD(T) correction term at the basis set limit (ΔCCSD(T)(limit)), which was estimated from the difference between the interaction energies calculated at the CCSD(T) and MP2 levels (ΔCCSD(T) = *E*_CCSD(T)_ − *E*_MP2_) by using the 6-31G* basis set [88,89]. Electrostatic energies were calculated as the interactions between distributed multipoles [90,91] of interacting molecules by using the ORIENT program [92]. Distributed multipoles up to hexadecapole were obtained on all atoms from the MP2/6-311G** level wave functions of isolated molecules by using the GDMA program [93]. Induction energies were calculated as the interactions of polarizable sites with the electric field produced by the distributed multipoles of the monomers [94]. The atomic polarizabilities of carbon (α = 10 a.u.), nitrogen (α = 8 a.u.), oxygen (α = 6 a.u.), fluorine (α = 3 a.u.), sulfur (α = 20 a.u.), and iodine (α = 34 a.u.) were used for the calculations [95]. The distributed multipoles were used only to estimate the electrostatic and induction energies. The MP2/6-311G** level optimized geometries of isolated molecules were used to calculate the intermolecular interaction energy potentials. The B3LYP calculations with Grimme’s D3 dispersion correction using the cc-pVTZ basis set (B3LYP-D3/cc-pVTZ) level interaction energy potentials [65]) were calculated with and without polarizable continuum model (PCM) [66] to evaluate the effects of the solvent. The B3LYP-D3 level interaction energies were calculated without BSSE correction.

## 4. Conclusions

In conclusion, we have studied the potential of SF_5_-substituted iodobenzenes as halogen bond donors. The simulated electrostatic potential values of SF_5_-substituted iodobenzenes, the ab initio molecular orbital calculations of intermolecular interactions with pyridine, and the ^13^C-NMR titration experiments of SF_5_-substituted iodobenzenes in the presence of halogen bond acceptors were investigated to assess the existence of halogen bonding. It should be noted that halogen bonding of iodobenzenes induced by the SF_5_-substituted group is strictly dependent on the position of SF_5_-substitution on the benzene ring, i.e., *o*-SF_5_-iodobenzene acts as halogen bond donor, while *m*- and *p*- SF_5_-iodobenzenes do not. The *ortho*-effect was also observed for a series of NO_2_-iodobenzenes. 3,5-Bis-SF_5_-iodobenzene is the most effective halogen bond donor in the series, and it is almost an equivalent to the well-known 3,5-bis-NO_2_-iodobenzene, as supported by our calculations and ^13^C-NMR titration experiments. These observations are in good agreement with the Mulliken charge of corresponding iodine. Since SF_5_-containing compounds are promising drug candidates, the present results should provide useful information for the rational design of drugs capable of halogen bonding with biomolecules. The X-ray crystallographic analyses of 3,5-bis-SF_5_-iodobenzene with halogen bond acceptors are now being considered

## Figures and Tables

**Table 1:** Electrostatic, induction, and dispersion energies of halogen-bonded complexes ^a^.

Complex	*E* (kcal mol^−1^)				
	*E*_int_^a^	*E*_es_^b^	*E*_ind_^c^	*E*_short_^d^	*E*_corr_^e^
*o*-SF_5_-iodobenzene---pyridine (**2a**)	−4.88	−4.48	−1.32	4.43	−3.52
*m*-SF_5_-iodobenzene---pyridine (**2b**)	−4.27	−4.20	−1.20	4.40	−3.27
*p*-SF_5_-iodobenzene---pyridine (**2c**)	−4.28	−4.02	−1.15	4.02	−3.13
3,5-bis-SF_5_-iodobenzene---pyridine (**2d**)	−5.21	−5.60	−1.65	5.54	−3.49
3,5-bis-NO_2_-iodobenzene---pyridine (**3**)	−4.83	−5.23	−1.52	4.46	−2.53
pentafluoro-iodobenzene---pyridine (**4**)	−5.71	−5.83	−1.68	5.11	−3.31
iodobenzene---pyridine (**5**)	−3.25	−2.73	−0.82	3.30	−2.99

^a^ Estimated interaction energy by coupled-cluster calculations with single and double substitutions with non-iterative triple excitations (CCSD(T)) at the basis set limit (*E*_CCSD(T)(limit)_). ^b^ Electrostatic energy. ^c^ Induction energy. ^d^ Contribution of short-range (orbital–orbital) interactions (= *E*_HF_ − *E*_es_ − *E*_ind_). The interaction energy by Hartree-Fock calculations with aug-cc-pVTZ basis set (HF/aug-cc-pVTZ) was used as *E*_HF_. *E*_short_ is mainly the contribution of exchange–repulsion interactions. ^e^ Contribution of electron correlation (= *E*_int_ − *E*_HF_). *E*_corr_ is mainly dispersion energy.

## Supplementary Materials

The following are available online. ^1^H, ^13^C, and ^19^F-NMR spectra of compounds **1**.
