# Supplementary material for: Studies of Halogen Bonding Induced by Pentafluorosulfanyl Aryl Iodides: A Potential Group of Halogen Bond Donors in a Rational Drug Design
Source: Molecules. 2019 Oct 7;24(19):3610. doi: 10.3390/molecules24193610 (PMC6803875; doi:10.3390/molecules24193610)
Supplement: Supplementary file 1 [file molecules-24-03610-s001.pdf]

Supplementary Materials

Studies of Halogen Bonding Induced by Pentafluorosulfanyl Aryl Iodides: A Potential Group of Halogen Bond Donors in a Rational Drug Design

Yuji Sumii [1], Kenta Sasaki [1], Seiji Tsuzuki [2] and Norio Shibata [1,3,*]

[1] Department of Nanopharmaceutical Sciences, and Department of Life Science and Applied Chemistry, Nagoya Institute of Technology, Gokiso, Showa-ku, Nagoya 466-8555, Japan; sumii.yuji@nitech.ac.jp (Y.S.); k.sasaki.699@stn.nitech.ac.jp (K.S.)

[2] Research Center for Computational Design of Advanced Functional Materials, AIST, Tsukuba, Ibaraki 305-8568, Japan; s.tsuzuki@aist.go.jp

[3] Institute of Advanced Fluorine-Containing Materials, Zhejiang Normal University, 688 Yingbin Avenue, 321004 Jinhua, China

* Correspondence: nozshiba@nitech.ac.jp; Tel./Fax: +81-52-735-7543

Table of contents

^{1}H, ^{19}F NMR, and ^{13}C spectra of compounds 2

^{1}H, ^{19}F NMR, and ^{13}C spectra of compounds

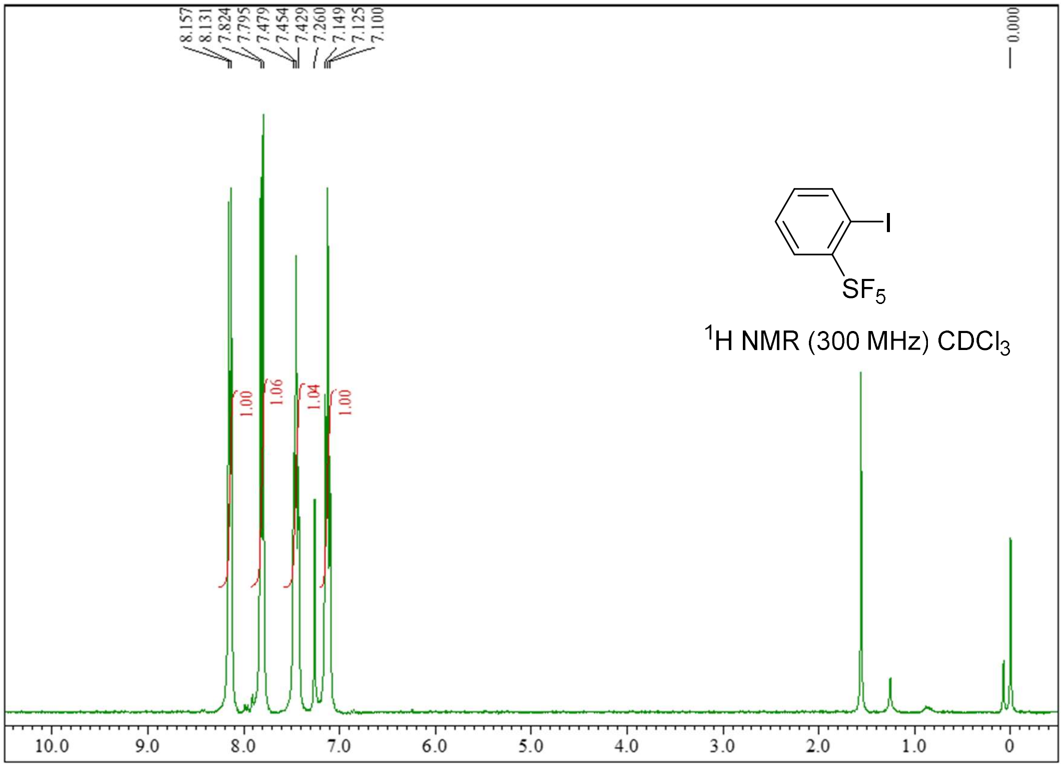

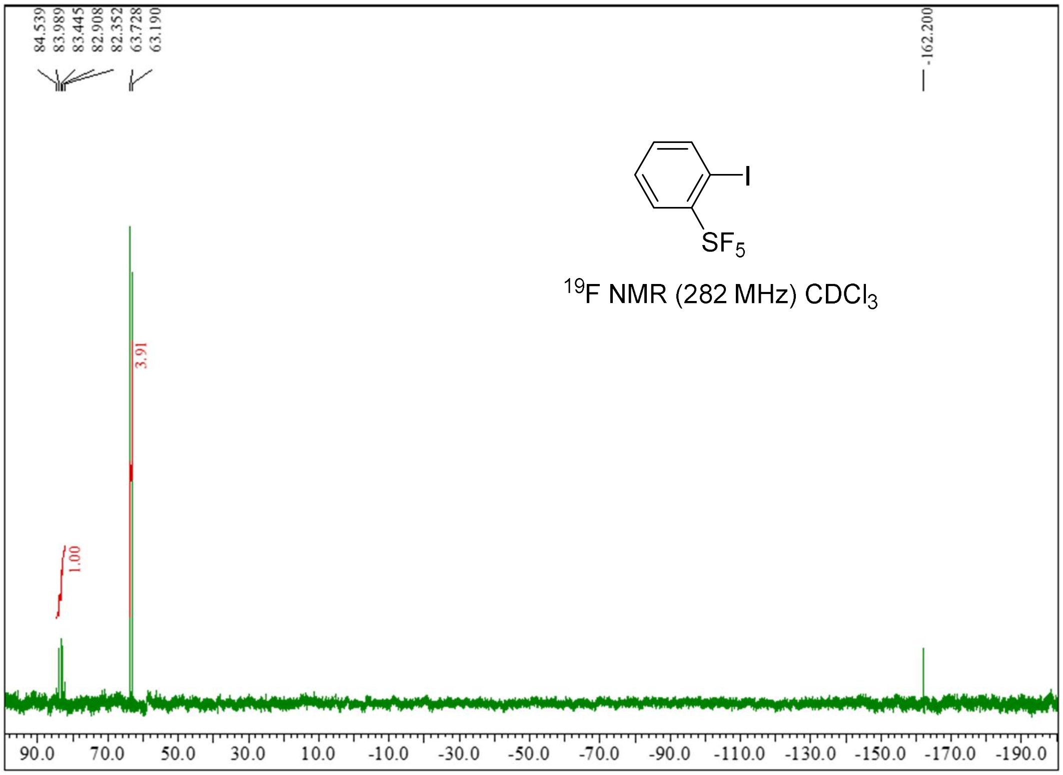

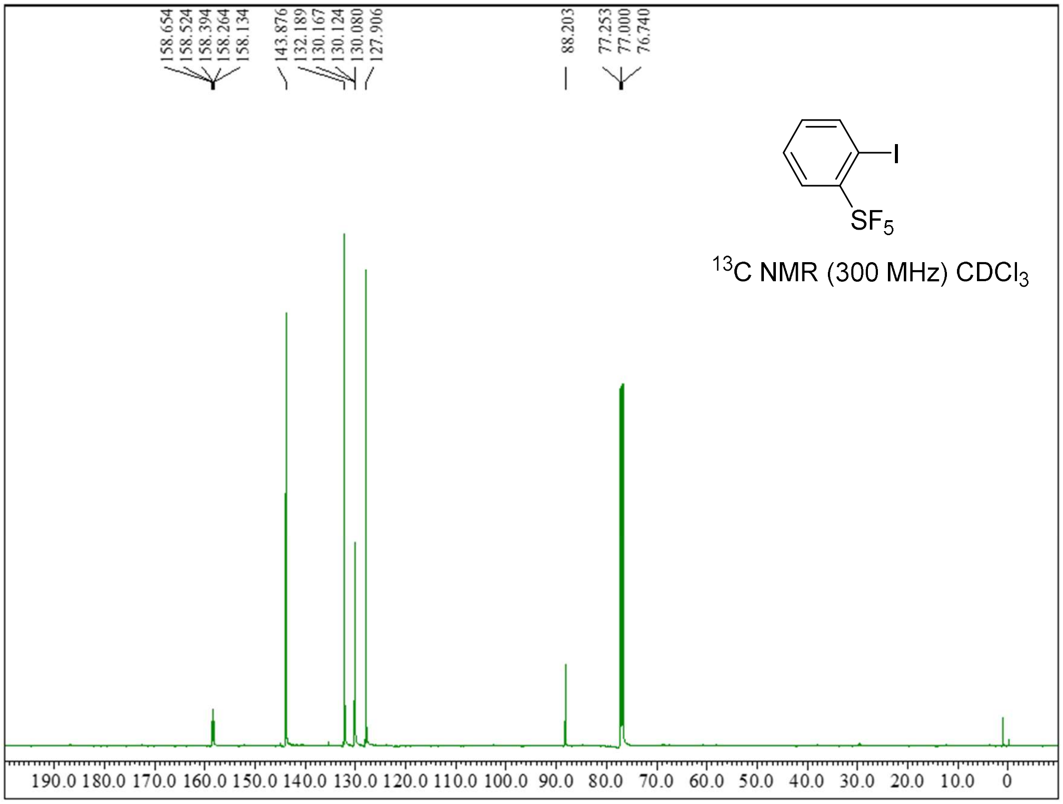
158.654
158.524
158.394
158.264
158.134
143.876
132.189
130.167
130.124
130.080
127.906
88.203
77.253
77.000
76.740
I
SF5
13C NMR (300 MHz) CDCl3
190.0 180.0 170.0 160.0 150.0 140.0 130.0 120.0 110.0 100.0 90.0 80.0 70.0 60.0 50.0 40.0 30.0 20.0 10.0 0

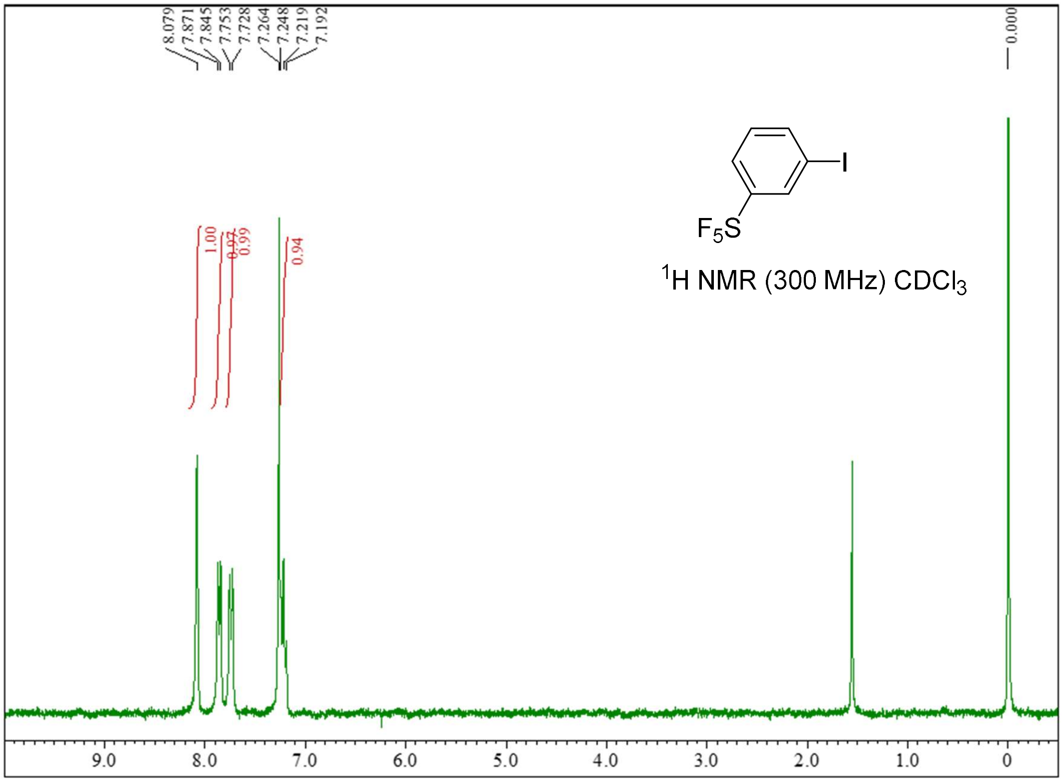
8.079
7.871
7.845
7.753
7.728
7.264
7.248
7.219
7.192
0.000
I
F5S
1.00
0.97
0.99
0.94
1H NMR (300 MHz) CDCl3
9.0 8.0 7.0 6.0 5.0 4.0 3.0 2.0 1.0 0

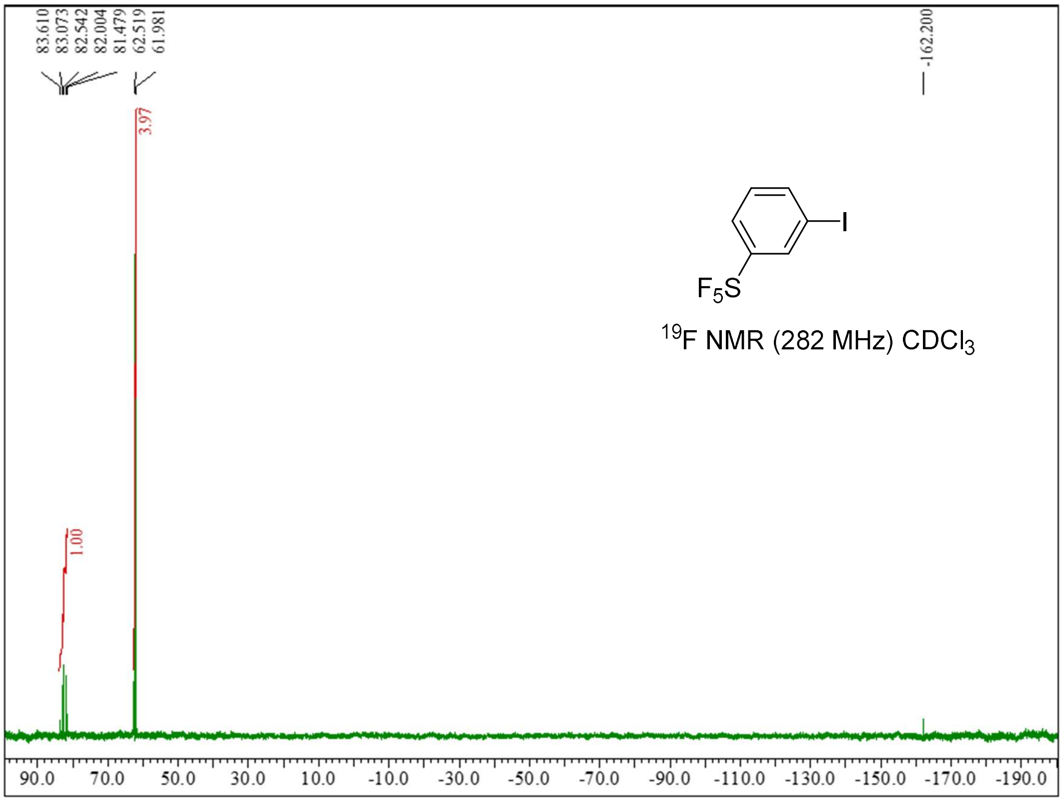
83.610
83.073
82.542
82.004
81.479
62.519
61.981
-162.200
3.97
1.00
F5S
I
19F NMR (282 MHz) CDCl3
90.0 70.0 50.0 30.0 10.0 -10.0 -30.0 -50.0 -70.0 -90.0 -110.0 -130.0 -150.0 -170.0 -190.0

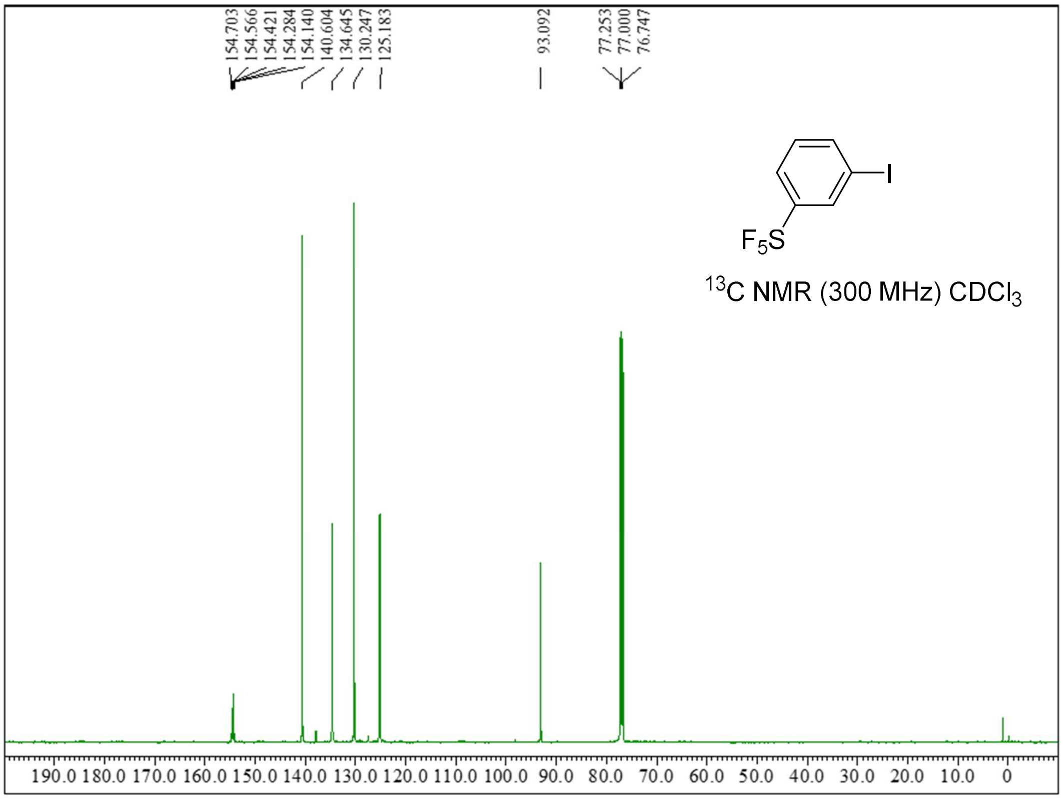
154.703
154.566
154.421
154.284
154.140
140.604
134.645
130.247
125.183
93.092
77.253
77.000
76.747
F5S
I
13C NMR (300 MHz) CDCl3
190.0 180.0 170.0 160.0 150.0 140.0 130.0 120.0 110.0 100.0 90.0 80.0 70.0 60.0 50.0 40.0 30.0 20.0 10.0 0

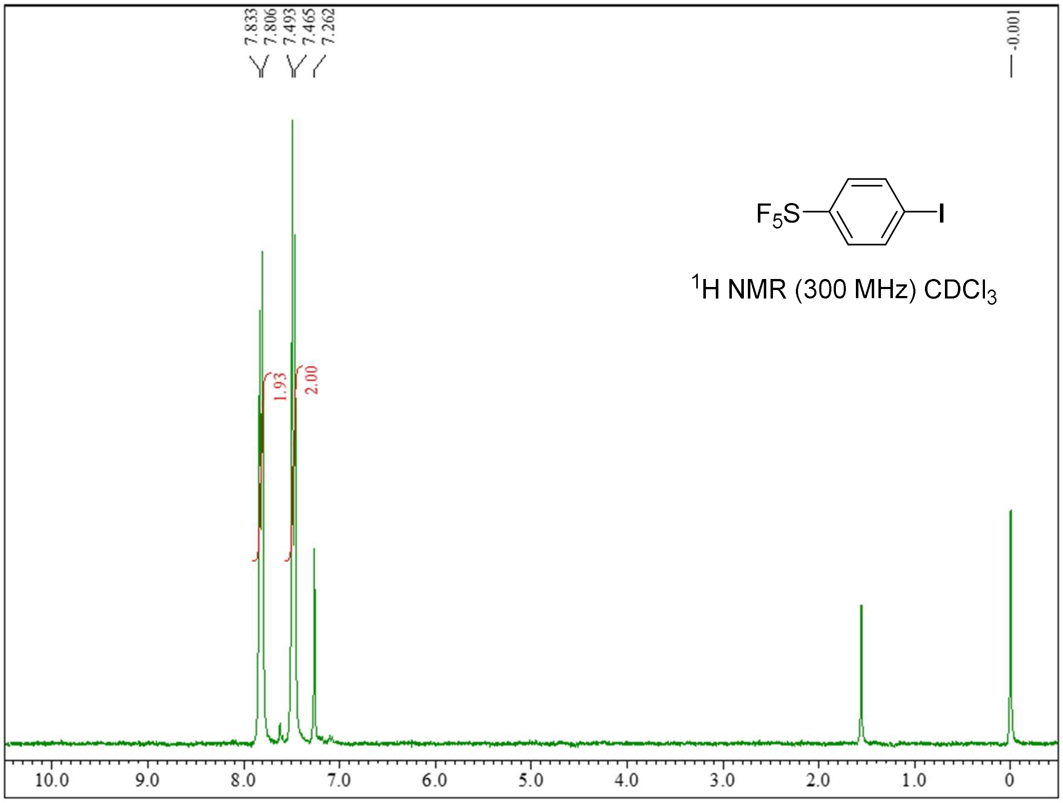
7.833
7.806
7.493
7.465
7.262
-0.001
1.93
2.00
F5S
I
1H NMR (300 MHz) CDCl3
10.0
9.0
8.0
7.0
6.0
5.0
4.0
3.0
2.0
1.0
0

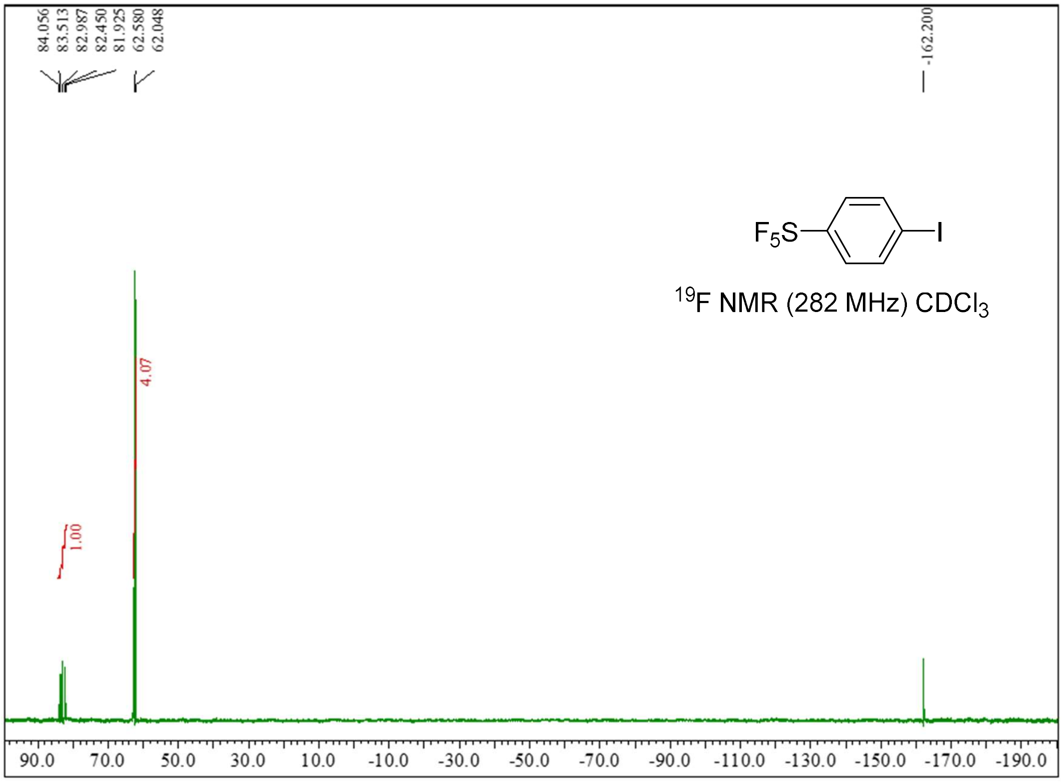
84.056
83.513
82.987
82.450
81.925
62.580
62.048
-162.200
F5S
I
19F NMR (282 MHz) CDCl3
4.07
1.00
90.0
70.0
50.0
30.0
10.0
-10.0
-30.0
-50.0
-70.0
-90.0
-110.0
-130.0
-150.0
-170.0
-190.0

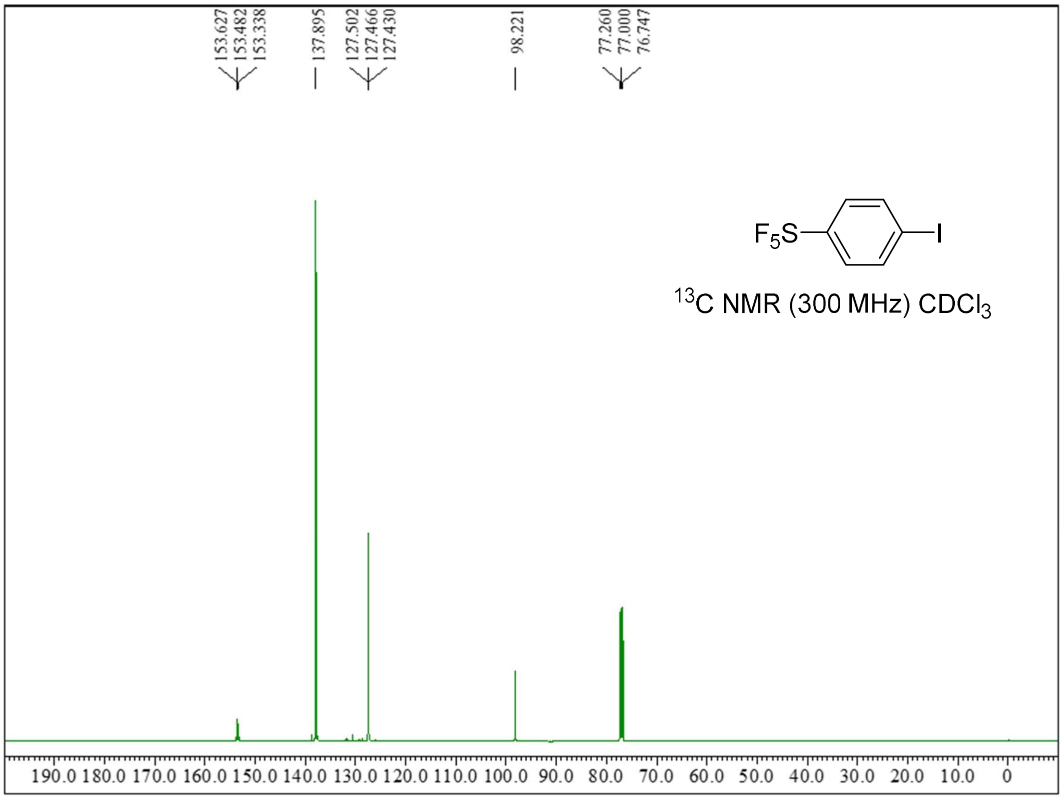
153.627
153.482
153.338
137.895
127.502
127.466
127.430
98.221
77.260
77.000
76.747
F_5S
I
^{13}C NMR (300 MHz) $CDCl_3$
190.0 180.0 170.0 160.0 150.0 140.0 130.0 120.0 110.0 100.0 90.0 80.0 70.0 60.0 50.0 40.0 30.0 20.0 10.0 0

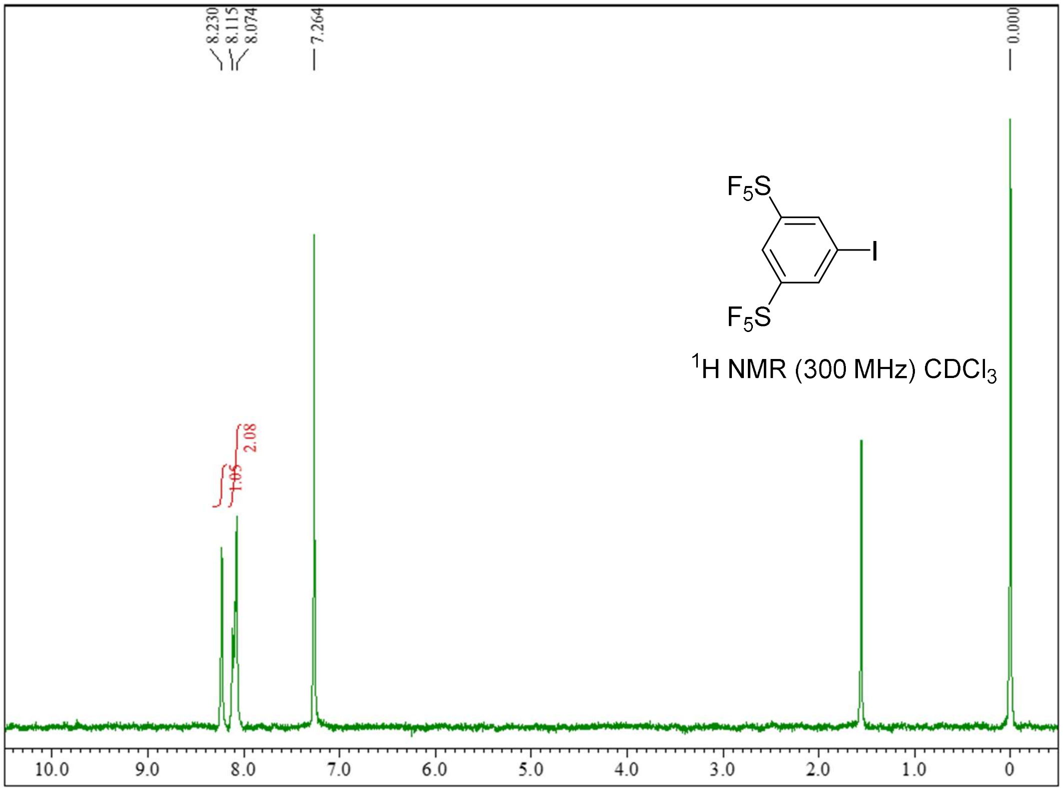
8.230
8.115
8.074
7.264
0.000
F_5S
I
F_5S
^{1}H NMR (300 MHz) $CDCl_3$
1.05
2.08
10.0 9.0 8.0 7.0 6.0 5.0 4.0 3.0 2.0 1.0 0

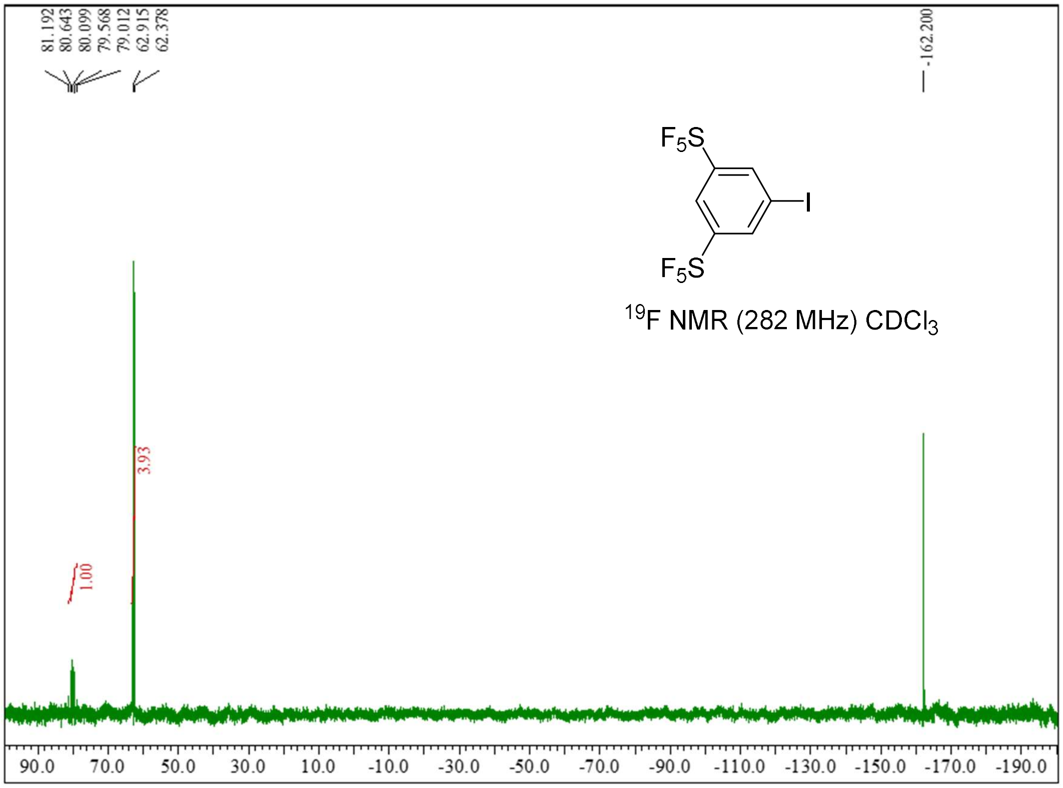
81.192
80.643
80.099
79.568
79.012
62.915
62.378
-162.200
F5S
I
F5S
19F NMR (282 MHz) CDCl3
3.93
1.00
90.0 70.0 50.0 30.0 10.0 -10.0 -30.0 -50.0 -70.0 -90.0 -110.0 -130.0 -150.0 -170.0 -190.0

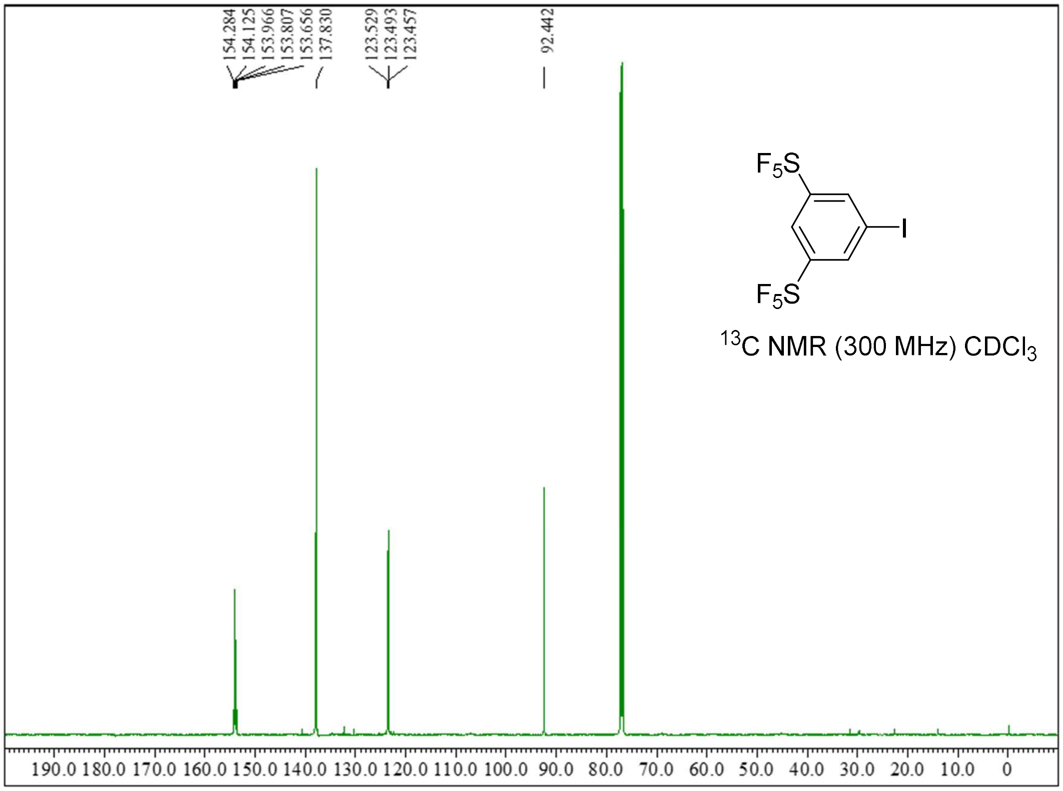
154.284
154.125
153.966
153.807
153.656
137.830
123.529
123.493
123.457
92.442
F5S
I
F5S
13C NMR (300 MHz) CDCl3
190.0 180.0 170.0 160.0 150.0 140.0 130.0 120.0 110.0 100.0 90.0 80.0 70.0 60.0 50.0 40.0 30.0 20.0 10.0 0
